# Constitutive Equation and Hot Compression Deformation Behavior of Homogenized Al–7.5Zn–1.5Mg–0.2Cu–0.2Zr Alloy

**DOI:** 10.3390/ma10101193

**Published:** 2017-10-18

**Authors:** Jianliang He, Datong Zhang, Weiweng Zhang, Cheng Qiu, Wen Zhang

**Affiliations:** National Engineering Research Center of Near-Net Shape Forming for Metallic Materials, South China University of Technology, Guangzhou 510640, China; mehejianliang@mail.scut.edu.cn (J.H.); mewzhang@scut.edu.cn (W.Z.); cqiu@scut.edu.cn (C.Q.); jack_eei@scut.edu.cn (W.Z.)

**Keywords:** aluminum alloy, hot compression, constitutive equation, microstructure

## Abstract

The deformation behavior of homogenized Al–7.5Zn–1.5Mg–0.2Cu–0.2Zr alloy has been studied by a set of isothermal hot compression tests, which were carried out over the temperature ranging from 350 °C to 450 °C and the strain rate ranging from 0.001 s^−1^ to 10 s^−1^ on Gleeble-3500 thermal simulation machine. The associated microstructure was studied using electron back scattered diffraction (EBSD) and transmission electron microscopy (TEM). The results showed that the flow stress is sensitive to strain rate and deformation temperature. The shape of true stress-strain curves obtained at a low strain rate (≤0.1 s^−1^) conditions shows the characteristic of dynamic recrystallization (DRX). Two Arrhenius-typed constitutive equation without and with strain compensation were established based on the true stress-strain curves. Constitutive equation with strain compensation has more precise predictability. The main softening mechanism of the studied alloy is dynamic recovery (DRV) accompanied with DRX, particularly at deformation conditions, with low Zener-Holloman parameters.

## 1. Introduction

7000 series Al–Zn–Mg–Cu alloys have a combination of high strength-to-density ratio, high hardness, and good resistance to stress corrosion, which have been widely used as structure materials in automotive and aerospace industries [1,2,3]. In order to reduce CO_2_ emission, light-weight cars have drawn great interests around the world. Application of high-strength aluminum alloys is an important way to reduce the weight of automobile. However, the limited workability is one of the main obstacles for extensive application of high-strength 7000 series alloys. In general, with the contents of alloying elements increasing, the strength of 7000 series alloys increase, and the workability turn worse. To improve the workability and plasticity of these alloys, methods such as composition optimization, grain refinement, and deformation processing optimization, etc., can be used.

Understanding the deformation behaviors of a newly-developed alloy is helpful for its processing design and optimization. A reliable constitutive equation containing stress, strain, strain rate, and deformation temperature can predict the flow stress and even microstructure evolution of the materials during plastic deformation. In general, three kinds of constitutive models can be employed to study the deformation behaviors of metallic materials, i.e., phenomenological, physical based, and artificial neural network (ANN) models [4,5]. Though phenomenological models lack of physical meaning, they are straightforward and widely used in the simulation of plastic deformation. Physical based models are more complicated since these models contain more material constants, but they offer an accurate description of deformation behavior and can be used in wide ranges of strain rate and temperature. ANN models can be used to solve the problems that cannot be settled by the above two methods, while the conditions for successful application are rigorous.

In recent years, a number of studies on flow stress behavior of aluminum alloys have been reported, in which the workability and microstructural evolution during plastic deformation at elevated temperature are investigated by isothermal hot compression tests. Chen et al. [6] investigated the effect of homogenization and solution treatment on the hot deformation behavior of 7085 aluminum alloy. Huang et al. [7] reported that the flow stress of 2026 aluminum alloy can be represented by the Zener—Holloman parameter *Z* in the hyperbolic sine equation with a hot deformation activation energy of 340.98 kJ/mol. Zhao et al. [8] optimized the practical extrusion of 6N01 aluminum alloy, based on numerical simulation with the obtained constitutive equation of the alloy. Generally, dynamic recovery (DRV) is the main softening mechanism of aluminum alloys, which are typical metallic materials with high stacking fault energy. However, some reports [9,10,11] have shown the occurrences of dynamic recrystallization (DRX) in aluminum alloys at low Z parameters, which correspond to high deformation temperatures and low strain rates. Therefore, to investigate the deformation behavior of a newly-developed alloy, establishing a constitutive equation and finding out the softening mechanism are fundamental works. 

In this paper, plastic deformation behavior of a newly-developed Al–Zn–Mg–Cu–Zr alloy with high Zn/Mg ratio and low Cu concentration was studied by hot compression tests on Gleeble-3500 thermal simulation machine. Two Arrhenius-typed constitutive equations without and with strain compensation were constructed based on the true stress-strain curves. The effect of deformation parameters on the flow behavior and microstructure, and the relationship between *Z* parameters and dynamic softening mechanism were investigated by electron back scattered diffraction (EBSD) and transmission electron microscopy (TEM) techniques.

## 2. Results and Discussion

### 2.1. True Stress-Strain Curves

**Figure 1:**
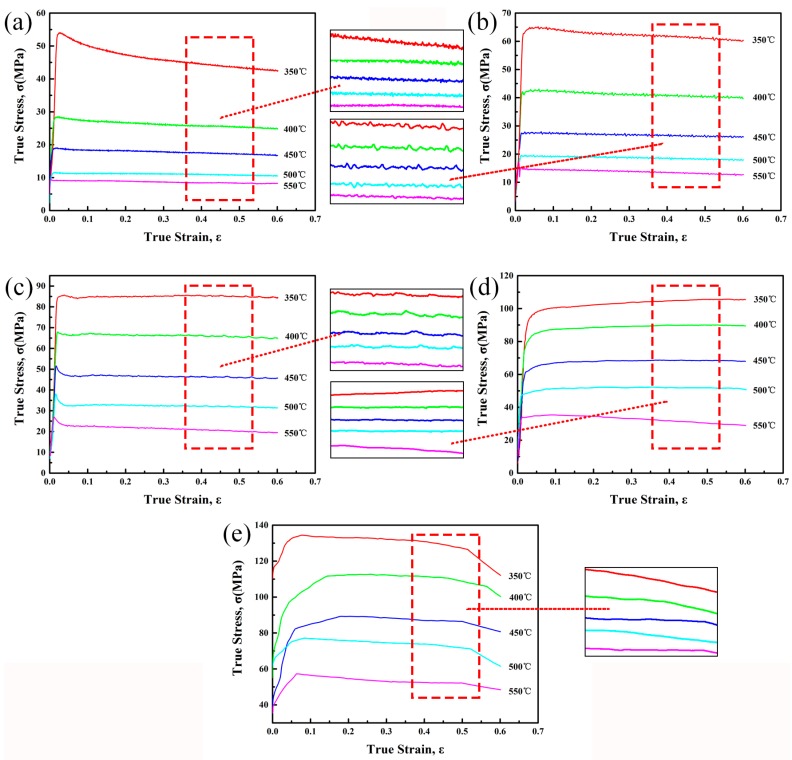
True stress–strain curves and magnified curves of the homogenized Al–7.5Zn–1.5Mg–0.2Cu–0.2Zr alloy with different deformation temperatures at various strain rates of (**a**) 0.001 s^−1^; (**b**) 0.01 s^−1^; (**c**) 0.1 s^−1^; (**d**) 1 s^−1^; and (**e**) 10 s^−1^.

Figure 1 shows a series of true stress-strain curves and their magnified curves obtained during hot compression tests of the experimental alloy. It can be found that the strain rate and deformation temperature have great influence on the flow stress level. Generally, the flow stress increases with increasing strain rate or decreasing deformation temperature. 

At the beginning of deformation, flow stress increases rapidly due to the significant work hardening effect that is caused by dislocation density increasing. As the deformation proceeds, the flow stress reaches a peak value and then decreases to a steady value during low strain rates (≤0.1 s^−1^) deformation. On the other hand, the flow stress reaches a steady state directly during high strain rates (1 s^−1^, 10 s^−1^) deformation. In addition, serrated curves can be found at low strain rates (≤0.1 s^−1^) conditions. These phenomena might be related to the occurrence of DRX. Generally, the main softening mechanism of high stacking fault energy materials, such as aluminum alloys, is DRV. However, some reports [9,10,11] shows that DRX occurs when the specimen deformed at low Z conditions. Therefore, in this work, the main dynamic softening mechanism might be DRV accompanied with DRX at a low strain rate (≤0.1 s^−1^) conditions, which would be further discussed combined with deformed microstructures later.

### 2.2. Constitutive Equation

Constitutive relation between flow stress (σ), strain rate ( $\dot{\varepsilon }$ ), and temperature (*T*) can be expressed by an Arrhenius type equation as follow [12,13,14,15]:

(1) $$\dot{\varepsilon }=AF\left(\sigma \right)\exp\left(-\frac{Q}{RT}\right)$$

where

F(σ)={(2)σn1              (ασ<0.8)(3)exp(βσ)          (ασ>1.2)(4)[sinh(ασ)]n         for all σ

where R is the universal gas constant (8.314 J·mol^−1^·K^−1^); *Q* is the activation energy of hot deformation; A, n_1_, α, β, and n are the material constants, α = β/n_1_.

By substituting the values of *F*(σ) into Equation (1) and then taking the natural logarithm of both sides, respectively, Equation (1) becomes:

lnε˙={(5)lnA−QRT+n1lnσ(6)lnA−QRT+βσ(7)lnA−QRT+nln[Sinh(ασ)]

**Figure 2:**
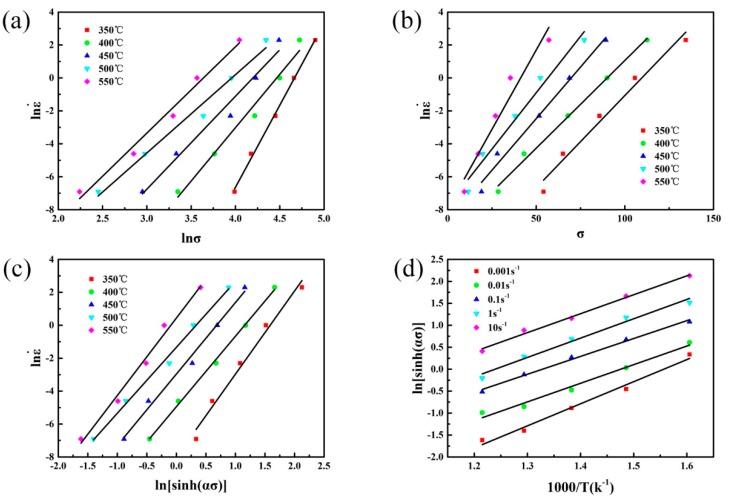
Relationship between (**a**) lnσ and ln $\dot{\varepsilon }$ ; (**b**) σ and ln $\dot{\varepsilon }$ ; (**c**) ln[sinh(ασ)] and $\ln\dot{\varepsilon }$ ; (**d**) 1000/T ; and ln[sinh(ασ)] .

In this work, the peak stress values are used to illustrate the calculation process of material constants in the Arrhenius type equation. The values of n_1_ and β are obtained from the mean slope values of lnσ–ln $\dot{\varepsilon }$ and σ–ln $\dot{\varepsilon }$ , as shown in Figure 2a,b, which is calculated to be 6.416 and 0.135 MPa^−1^, respectively. Thus, the value of α = β/n_1_ = 0.021 MPa^−1^. When compared with other researches on Al–Zn–Mg–Cu alloys [16,17,18], the value of this work is at the same level.

In order to calculate the activation energy *Q* of hot deformation, Equation (7) is differentiated as follows:

(8) $$Q=R{\left[\frac{\partial \ln\dot{\varepsilon }}{\partial ln\left[\sin h\left(\alpha \sigma \right)\right]}\right]}_{T}{\left[\frac{\partial \ln\left[\sin h\left(\alpha \sigma \right)\right]}{\partial \left(1/T\right)}\right]}_{\dot{\varepsilon }}$$

The values of $\partial \ln\dot{\varepsilon }/\partial \ln\left[\sin h\left(\alpha \sigma \right)\right]$ and ∂ln[sinh(ασ)]/∂(1/T) are obtained from the mean slope values of $\ln[\sin h\left(\alpha \sigma \right)]-\ln\dot{\varepsilon }$ and 1000/T−ln[sinh(ασ)] , as shown in Figure 2c and Figure 2d, which is calculated to be 4.474 and 4.411, respectively. Thus, the deformation activation energy *Q* of the studied alloy is 164.075 kJ·mol^−1^, which is close to that of 7050 aluminum alloy (160.3 kJ·mol^−1^), as reported by Deng et al. [19]. However, when compared with the 7150 aluminum alloy (229.75 kJ·mol^−1^) reported by Jin et al. [20] and the 7075 aluminum alloy (269.04 kJ·mol^−1^) reported by Lin et al. [21], the *Q* value of the studied alloy is relatively low. It indicates that the deformation activation energy is sensitive to the alloy compositions of the materials. According to Equation (1), at the same deformation temperature and strain rate, flow stress increases with increasing *Q* value, which means that materials with lower *Q* value can be deformed more easily with a lower force. From this point of view, the formability is relatively good as compared with the 7000 series alloys with high *Q* values mentioned above. Besides, initial microstructures have distinct influence on the values of *Q*, as reported by Chen et al. [6]. The *Q* value of the studied alloy is higher than the self-diffusion activation energy of pure aluminum (142 kJ·mol^−1^) [22], indicating that DRX might occur in this alloy during hot compression deformation.

In addition, the effects of strain rate and temperature on deformation behavior can also be represented by the Zener–Holloman parameter (noted as *Z* parameter):

(9) $$Z=\dot{\varepsilon }\exp\left(\frac{Q}{RT}\right)=A{\left[\sin h\left(\alpha \sigma \right)\right]}^{n}$$

Taking the natural logarithm of both sides:

(10) lnZ=lnA+nln[sinh(ασ)]

**Figure 3:**
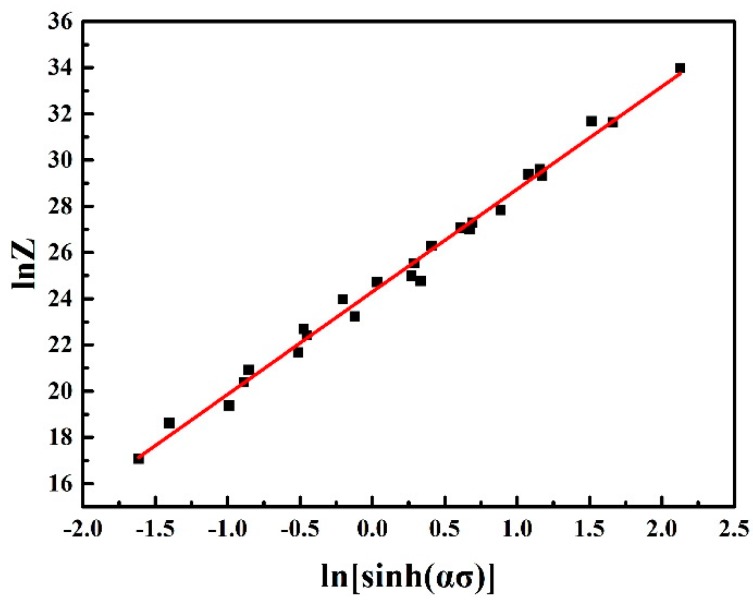
Relationship between ln*Z* and ln[sinh(ασ)].

The values of *Z* parameter can be calculated by substituting strain rates, deformation temperature, and value of *Q* into Equation (9). The relationship between ln*Z* and ln[sinh(ασ)] is showed in Figure 3. The values of n and lnA are the slope and intercept of the fitting straight line, which is calculated to be 4.44 and 24.302, respectively. Thus, the value of A is 3.58 × 10^10^.

Finally, substituting the value of α, *Q*, n and A into Equation (1), the constitutive equation is as follow:

(11) $$\dot{\varepsilon }=3.58\times 10^{10}{\left[\sin h\left(0.021\sigma \right)\right]}^{4.44}\exp\left(-\frac{1.641\times 10^{5}}{RT}\right)$$

Moreover, the flow stress can be written as a function of *Z* parameter:

(12) $$\sigma =\frac{1}{0.021}\ln\left\{{\left(\frac{Z}{3.58\times 10^{10}}\right)}^{1/4.44}+{\left[{\left(\frac{Z}{3.58\times 10^{10}}\right)}^{2/4.44}+1\right]}^{1/2}\right\}$$

### 2.3. Compensation of Strain

**Figure 4:**
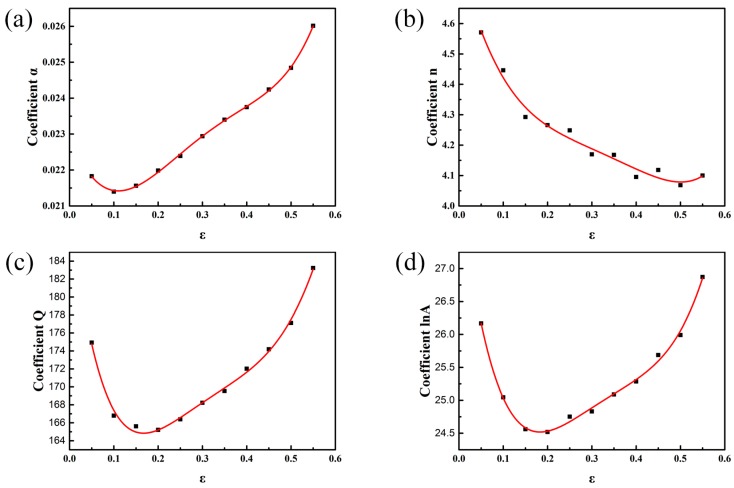
Relationship between strain and material constants of (**a**) α; (**b**) n; (**c**) *Q*; and (**d**) lnA.

From the true stress-strain curves shown in Figure 1, it can be observed that the flow stress changes with the increasing strain. However, according to the constitutive equation above, the flow stress only depends on the deformation temperature and strain rate. Thus, in order to achieve a more accurate flow stress prediction, the influence of strain should be taken into account, which can be expressed by using polynomial functions [23,24,25]. To establish the constitutive equation with strain compensation, the material constants (α, *Q*, n, A) at different strains ranging from 0.05 to 0.55 with the interval of 0.05 are evaluated. It can be considered that a fifth order polynomial function can well represent the influence of strain on material constants, as shown in Equations (13)–(16). Values at different strains and a fitting curve of each material constant are shown in Figure 4, and the coefficients of the polynomial functions are given in Table 1. Therefore, the flow stress at a certain strain can be predicted by evaluating the material constants at this strain.

(13) $$\alpha =B_{0}+B_{1}\varepsilon +B_{2}\varepsilon^{2}+B_{3}\varepsilon^{3}+B_{4}\varepsilon^{4}+B_{5}\varepsilon^{5}$$

(14) $$n=C_{0}+C_{1}\varepsilon +C_{2}\varepsilon^{2}+C_{3}\varepsilon^{3}+C_{4}\varepsilon^{4}+C_{5}\varepsilon^{5}$$

(15) $$Q=D_{0}+D_{1}\varepsilon +D_{2}\varepsilon^{2}+D_{3}\varepsilon^{3}+D_{4}\varepsilon^{4}+D_{5}\varepsilon^{5}$$

(16) $$\mathrm{lnA}=E_{0}+E_{1}\varepsilon +E_{2}\varepsilon^{2}+E_{3}\varepsilon^{3}+E_{4}\varepsilon^{4}+E_{5}\varepsilon^{5}$$

### 2.4. Verification of Constitutive Equation

**Figure 5:**
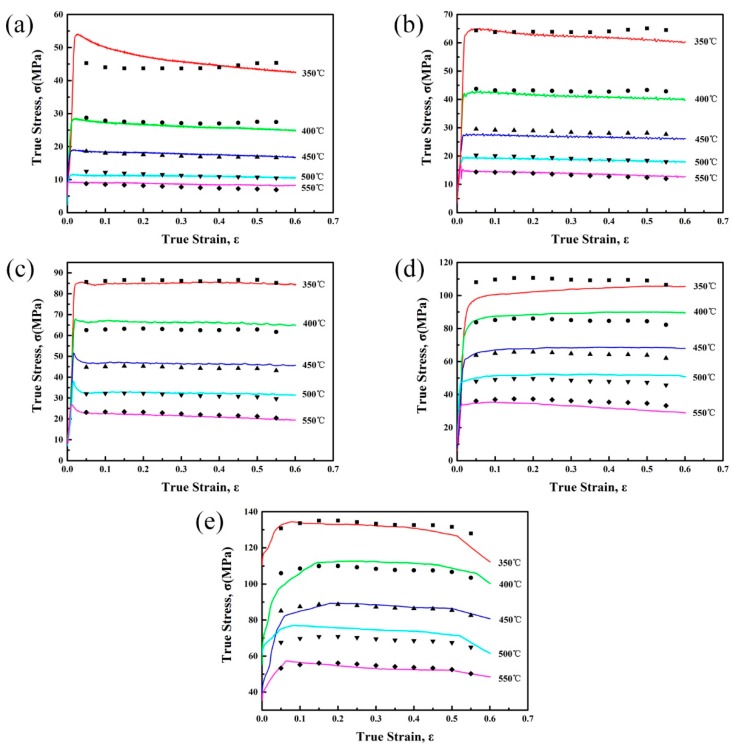
Comparison between flow stress calculated from constitutive equation with strain compensation (symbols) and experimental data (curves). (**a**) 0.001 s^−1^; (**b**) 0.01 s^−1^; (**c**) 0.1 s^−1^; (**d**), 1 s^−1^ and (**e**) 10 s^−1^.

Figure 5 shows the comparison between predicted and experimental values of flow stress, which indicates that the constitutive equation with compensation of strain has good agreement with the experimental data. 

Correlation coefficient (*R*) and average absolute relative error (*AARE*) [26,27,28] are evaluated to further verify the accuracy of the developed constitutive equation, which are shown as follows:

(17) $$R=\frac{\sum_{i=1}^{N}\left(E_{i}-\bar{E}\right)\left(P_{i}-\bar{P}\right)}{\sqrt{\sum_{i=1}^{N}{\left(E_{i}-\bar{E}\right)}^{2}\sum_{i=1}^{N}{\left(P_{i}-\bar{P}\right)}^{2}}}$$

(18) $$AARE=\frac{1}{N}\overset{N}{\underset{i=1}{\sum }}\left|\frac{E_{i}-P_{i}}{E_{i}}\right|\times 100$$

where *E_i_* and *P_i_* are the experimental data and the predicted value, respectively; $\bar{E}$ and $\bar{P}$ are the mean values of *E_i_* and *P_i_*, respectively; *N* is the total number of data in the rearch. By calculating, the values of *R* and *AARE* of constitutive equation without strain compensation are 0.9946% and 8.39%, respectively. On the other hand, for constitutive equation with strain compensation, the values of *R* and *AARE* are 0.9953% and 4.90%, respectively. Therefore, to have more accurate predictability, the influence of strain should be considered in establishment of constitutive equations.

### 2.5. Microstructure Evolution

**Figure 6:**
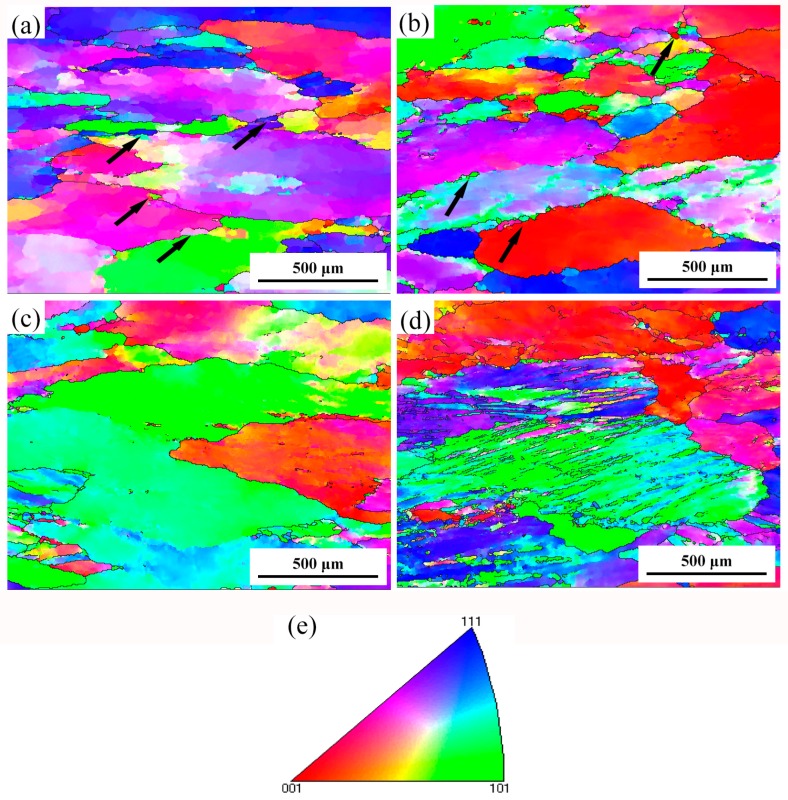
Microstructures of the specimens deformed under different parameters. (**a**) 500 °C/0.01 s^−1^; (**b**) 450 °C/0.1 s^−1^; (**c**) 450 °C/1 s^−1^; (**d**) 450 °C/10 s^−1^, and (**e**) representation of the color code used to identify the crystallographic orientations on standard stereographic projection (red: [0 0 1]; blue: [1 1 1]; green: [1 0 1]).

**Figure 7:**
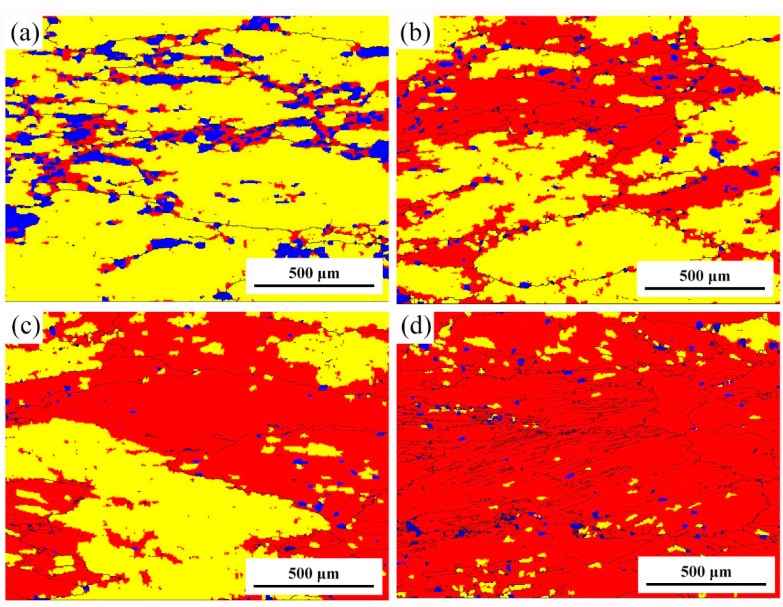
Regions with different misorientation angles in the deformed microstructures under different parameters. (**a**) 500 °C/0.01 s^−1^ (ln*Z* = 20.9); (**b**) 450 °C /0.1 s^−1^ (ln*Z* = 25); (**c**) 450 °C/1 s^−1^ (ln*Z* = 27.3); and, (**d**) 450 °C /10 s^−1^ (ln*Z* = 29.6).

EBSD figures of the specimens deformed under different conditions are shown in Figure 6. When compared with the initial grains shown in Figure 9, most of the grains were elongated after compression deformation. As shown in Figure 7a,b, new small equiaxial grains (shown by arrows) can be found at serrated grains boundaries at low strain rates deformation conditions, indicating the occurrence of DRX. Haghdadi et al. [29] reports that the softening mechanism of high stacking fault energy materials in low strain rate conditions is continous dynamic recrystallization (CDRX). Therefore, it is considered that the new small grains are formed through CDRX mechanism in the studied alloy. On the other hand, for high-strain-rate deformed specimens as shown in Figure 7c,d, the microstructures are heterogeneous and obvious adiabatic shear bands can be found in the specimen deformed at 450 °C/10 s^−1^. During high strain rates deformation, there is insufficient time for conducting of heat generated, which induces localized flow and shear band deformation. Moreover, DRX grains can be hardly found in Figure 7c,d. Haghdadi reported high strain rate will trigger discontinuous DRX (DDRX) in α + γ two-phase steel [29]. Since our material is not a two-phase alloy, DDRX is not observeed in the specimens deformed at high Z conditions.

Figure 7 shows regions with different misorientation angles in the deformed microstructures under different parameters. Regions in different colors represent different misorientation angles with the neighbouring grains or structures, in which blue represents misorientation angles >7.5°. The fraction of blue region for ln*Z* = 20.9, 25, 27.3, 29.6 are 12.6%, 2.8%, 1.2%, 2.1%, respectively. Obviously, fraction of blue region in Figure 7a is much higher than other specimens. The blue regions are distributed along the grain boundaries, corresponding to the new small grains in Figure 7a, which resulted from the occurrence of DRX. In addition, the fraction of blue region in Figure 7b is a little higher than the rest two specimens, as shown in Figure 7c,d. This phenomenon means that 450 °C/0.1 s^−1^ (ln*Z* = 25) might be or near the critical condition for the occurrence of DRX. On the other hand, the fraction of yellow region (1°–7.5°, 177.1%/ln*Z* = 20.9, 57.3%/ln*Z* = 25, 41.9%/ln*Z* = 27.3, 5.6%/ln*Z* = 29.6) also increases with the decreasing *Z* value. During continuous DRX in high stacking fault energy metals, new grains were form by progressive increase of low angle boundary misorientation [25]. When the specimens are compressed at high deformation temperatures and low strain rates, there is sufficient energy and time for glide and climb of dislocations, which can promote the occurrences of DRV and DRX. Because of the better softening effect of DRX, resistance to deformation of the studied alloy decreases, which appear as obvious decline of true stress. Therefore, combining the true stress-strain curves and microstructure examination, the main softening mechanism of the studied alloy is DRV accompanied with DRX, particularly at low *Z* deformation conditions.

**Figure 8:**
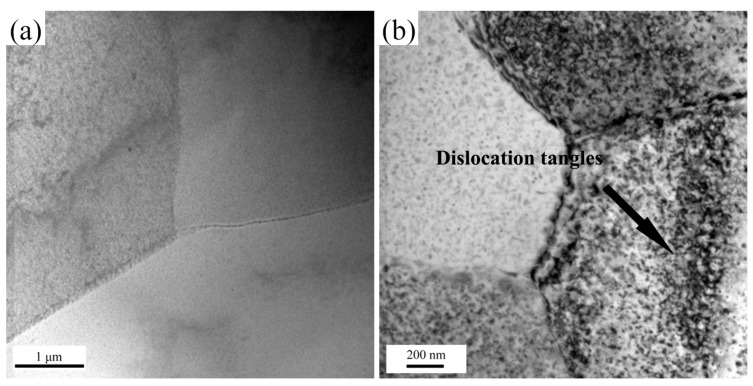
Transmission electron microscopy (TEM) microstructures of specimens deformed under different parameters. (**a**) 500 °C/0.01 s^−1^ (ln*Z* = 20.9); (**b**) 450 °C/1 s^−1^ (ln*Z* = 27.3).

TEM microstructures of the studied alloy deformed under different conditions are show in Figure 8. At low Z deformation condition (ln*Z* = 20.9), the dislocations density is at low level, indicating tha DRX took place to form new grains. While at a higher Z deformation condition (ln*Z* = 27.3), obvious dislocation tangles can be found and the subgrain boundaries become indistinct. During high strain rates deformation, though the stored distortion energy increase rapidly, there is no sufficient time for migration of grain boundaries, so it is hardly to observe DRX grains in the high Z specimens.

## 3. Materials and Methods

**Figure 9:**
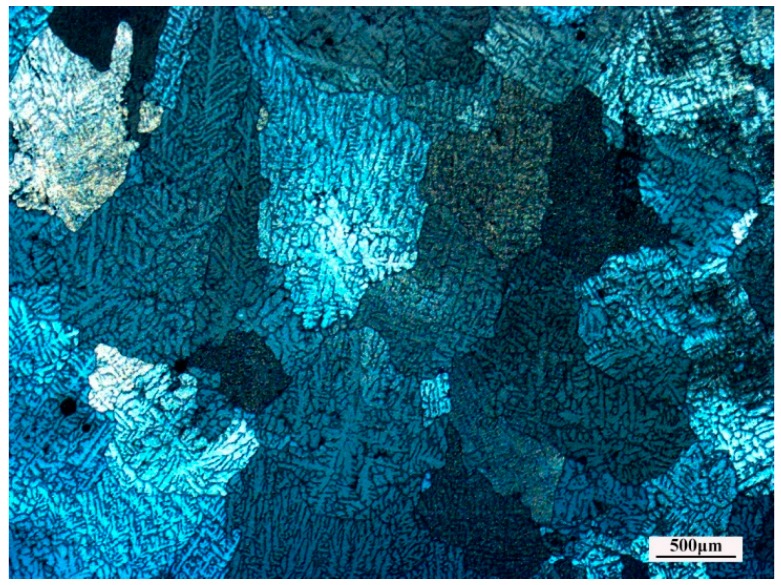
Optical microstructure of the as-homogenized Al–7.5Zn–1.5Mg–0.2Cu–0.2Zr alloy.

The chemical composition of the as-cast Al–Zn–Mg–Cu–Zr alloy used in the present work is shown in Table 2. This alloy was prepared by metal mold casting and then homogenized at 470 °C for 24 h. The optical micrograph of the homogenized microstructure is shown in Figure 9. Coarse equiaxial grains can be seen and the average grain size is about 500 μm. 

Cylindrical specimens of 15 mm in height and 10 mm in diameter for hot compression tests were machined from the center of the ingot. The hot compression tests were carried out on Gleeble-3500 thermal simulation machine (DSI, New York, NY, USA). The experimental temperatures ranged from 350 °C to 550 °C, and the strain rates ranged from 0.001 s^−1^ to 10 s^−1^. Each specimen was heated to the deformation temperature at the heating rate of 100 °C/min and held for 2 min before compress test. The specimens were compressed to 60% reduction and then quenched in water immediately. The deformed specimens for EBSD investigation were sliced parallel to the axial section and the cut surface was mechanically polished using standard techniques and electrochemically polished in a solution containing 5 mL HClO_4_ and 95 mL alcohol at 20 V for 20 s at room temperature. EBSD investigations were carried out on a S-3400N scanning electron microscope (Hitachi, Tokyo, Japan). TEM observation was carried out on a JEM-2200FS transmission electron microscope (JEOL, Tokyo, Japan) and specimens were prepared by mechanical polish followed by twin-jet electro-polishing, using 30% nitric acid +70% methanol solution. 

## 4. Conclusions

In this study, plastic deformation behavior of homogenized Al–7.5Zn–1.5Mg–0.2Cu–0.2Zr alloy was studied by hot compression tests. The main conclusions are as follows:

(1) The flow stress of the studied alloy increases with a decreasing deformation temperature or an increasing strain rate. Serrate true stress-strain curves are obtained at low strain rates (≤0.1 s^−1^) conditions, owing to the occurrence of DRX.
(2) The deformation activation energy *Q* is 164.075 kJ·mol^−1^. Two constitutive equations without and with strain compensation are established. The constitutive equation with strain compensation can predict the flow stress more accurately with the *R* and *AARE* values of 0.9953 and 4.90%, respectively.
(2) Obvious shear bands exit in the specimens deformed at high *Z* conditions, while specimens deformed at low *Z* conditions exhibit a more homogeneous microstructure. The main softening mechanism of the studied alloy is DRV accompanied with DRX, particularly at low *Z* deformation conditions.

## Figures and Tables

**Table 1:** Coefficients of polynomial fitting curves for material coefficients.

α		n		*Q*		lnA	
**B_0_**	0.02298	**C_0_**	4.81256	**D_0_**	190.25642	**E_0_**	28.35338
**B_1_**	−0.03302	**C_1_**	−5.71977	**D_1_**	−417.04791	**E_1_**	−57.06825
**B_2_**	0.21595	**C_2_**	21.87165	**D_2_**	2439.5926	**E_2_**	299.59736
**B_3_**	−0.43515	**C_3_**	−39.33363	**D_3_**	−6262.72669	**E_3_**	−669.06005
**B_4_**	0.22117	**C_4_**	19.28741	**D_4_**	7391.72494	**E_4_**	627.70979
**B_5_**	0.15942	**C_5_**	11.82051	**D_5_**	−2984.10256	**E_5_**	−136.35385

**Table 2:** The chemical composition of the experimental alloy (wt %)

Zn	Mg	Cu	Zr	Fe	Si	Al
7.65	1.57	0.16	0.17	0.05	0.03	Balance
